# Recent Advances in Randomness Extraction

**DOI:** 10.3390/e24070880

**Published:** 2022-06-26

**Authors:** Eshan Chattopadhyay

**Affiliations:** Department of Computer Science, Cornell University, Ithaca, NY 14850, USA; eshan@cs.cornell.edu

**Keywords:** randomness extractor, non-malleability, explicit construction

## Abstract

The area of randomness extraction has seen interesting advances in recent years, with rapid progress on many longstanding open problems, along with the introduction of many new notions that played a key role in this development. We survey this progress and highlight new definitions and notions that have been the subject of intense study in recent work.

## 1. Introduction

Randomness is a powerful resource in computer science and is widely used in most areas of computer science, such as algorithm design, cryptography, distributed computing, sampling, etc. There are central computational problems, such as the factorization of multivariate polynomials, for which we have efficient randomized algorithms but no deterministic algorithm is known yet (for more examples of randomized algorithms, we refer the reader to an excellent book on this topic [1]). Further, randomness is crucial in the field of cryptography, and in fact it is known that the quality of randomness (formalized below) is required to be very high for these applications to be secure [2].

This widespread use of randomness has lead to intense theoretical studies about the use of randomness in computational applications. Central questions in this area are of the following two flavors: (i) Is randomness fundamentally required for efficient algorithm design? (ii) How do we generate high-quality randomness for applications (which is crucially required in areas such as cryptography and distributed computing)?

While it seems that many fundamental problems in algorithm design are efficiently solvable only if we allow the algorithm to use random bits, it is now widely believed that randomness may not be intrinsically required for efficient computation. This stems from evidence [3,4] from computational complexity theory that proves that every efficient randomized algorithm can be *derandomized* to an efficient deterministic algorithm under very plausible conjectures in complexity theory. This is a sharp contrast to other areas of computer science such as cryptography, where, as we noted above, high-quality random bits are necessary for even basic protocols.

The focus of this article is targeted towards the latter applications, where it is of vital importance to ensure that the randomness used is of high quality. We will study these questions through the lens of theoretical computer science, where the investigations are theoretical in nature, and we will insist on mathematical guarantees about the quality of randomness that is being used.

It is widely known that the sources of randomness used in practice are defective in general, and only produce distributions that contain some amount of entropy (see [5] for some discussion on various means of collecting randomness from physical phenomena). This motivates the area of randomness extraction, where the central goal is to purify defective sources of randomness to produce truly random bits. Informally, an extractor Ext is simply a (deterministic) algorithm that takes as input a sample *x* from a distribution *X*, and outputs Ext(x). Thus, the output distribution of the extractor is given by Y=Ext(X), and ideally one would like to guarantee that if *X* itself contained some amount of randomness (measured in terms of entropy), then the output distribution *Y* is (almost) purely random.

The early work of von Neumann [6] in fact considered the problem of randomness extraction in the simple setting wherein a stream of independent, biased bits are supplied to the extractor and gave an elegant extractor for such sources. This extractor is used in the design of Intel’s TRNG (true random number generator) [7]. Another recent example of a practical randomness extractor was given in [8], where the authors presented efficient means of prodcing true randomness from big data.

We now formalize the intuitions developed above, and introduce a very general method of measuring the quality of a source and investigate the problem of randomness extraction from a theoretical perspective. The most widely used model of a weak source is based on the notion of min-entropy [9,10]. For a source (distribution) *X*, we define its min-entropy as $H_{\infty }\left(X\right)=\min_{x\in support(X)}\left\{\log\left(1/\mathrm{Pr}\left[X=x\right]\right)\right\}$. This can be considered a worst-case analogue of the more popularly used notion of Shannon entropy. (In the setting of extractors, it turns out that the notion of min-entropy is better suited.)

We use the notion of an (n,k)-source, which is a distribution on ${\left\{0,1\right\}}^{n}$ with min-entropy at least *k*. We note that the min-entropy parameter *k* can range from 0 to *n*. Another means of viewing an (n,k)-source *X* is that we are given the guarantee that the maximum weight placed by XX on any point in its support is bounded by $2^{-k}$. For example, a distribution which is flat (i.e., uniform) on a subset $A\subset {\left\{0,1\right\}}^{n}$ of cardinality $2^{k}$ is an (n,k)-source.

We are now ready to define randomness extractors more formally.

We first recall the definition of statistical distance that is used to measure the quality of the output of the extractor. For distributions X,Y on some universe Ω, define the statistical distance $|X-Y|:=\frac{1}{2}\cdot \sum_{w\in \Omega }\left|\mathrm{Pr}\left[X=w\right]-\mathrm{Pr}\left[Y=w\right]\right|$.

**Definition** **1** (Extractor)**.** *Let X be a family of distributions supported on ${\{0,1\}}^{n}$. We say that a function ${\mathrm{Ext}:\{0,1\}}^{n}{\to \{0,1\}}^{m}$ is an extractor for X with error ϵ if the following holds: for any distribution X∈X, we have $|\mathrm{Ext}(X)-U_{m}|\leq \epsilon$, where $U_{m}$ denotes the uniform distribution on m bits.*

We note that the guarantee that the distribution Ext(X) being ϵ-close to the uniform distribution (i.e., the statistical distance) is a very strong one in the following sense: it can be shown that for any adversary $A{:\{0,1\}}^{m}\to \{0,1\}$ trying to distinguish Ext(X) from the uniform distribution $U_{m}$, it has an advantage of, at most, ϵ; more formally, we have the guarantee $|\mathrm{Pr}\left[A\left(Ext\left(X\right)\right)=1\right]-\mathrm{Pr}\left[A\left(U_{m}\right)=1\right]|\;\leq \;\epsilon$. Note that this guarantee does not assume the computational limitation of the adversary A.

Given the above definitions, it is a natural question to construct randomness extractors. Unfortunately, a folklore observation rules out the existence of an extractor for the class of (n,n−1)-sources. This can be seen as follows: suppose, if possible, that ${\mathrm{Ext}:\{0,1\}}^{n}\to \{0,1\}$ is such an extractor (with error <1/2). Define the sets $S_{b}=\left\{x{\in \{0,1\}}^{n}:\mathrm{Ext}(x)=b\right\}$, for b=0,1. Clearly, the cardinality of at least one of $S_{0}$ and $S_{1}$ is $2^{n-1}$. Say $|S_{0}|\geq 2^{n-1}$. Thus, one arrives at a contradiction by considering the distribution $X_{0}$ that is uniform on $S_{0}$, since $X_{0}$ has min-entropy at least n−1 and Ext(x)=0 for all $x\in S_{0}$.

To circumvent this difficulty, Nisan and Zuckerman [11] introduced the notion of a seeded extractor. Informally, a seeded extractor is supplied with an additional independent short seed (which is independent of the defective source) to extract randomness from the weak source. We now introduce this notion more formally.

**Definition** **2** (Seeded Extractor)**.** *An (n,k)-seeded extractor ${\mathrm{Ext}:\{0,1\}}^{n}\times {\{0,1\}}^{d}{\to \{0,1\}}^{m}$ with seed length d and error ϵ satisfies, for any (n,k)-source X, $|Ext\left(X\right)-U_{m}|\leq \epsilon$. Further, we say that *Ext* is a strong-seeded extractor if $|\left(Ext\left(X,U_{d}\right)\right),U_{m}-\left(U_{d},U_{m}\right)|\leq \epsilon$.*

Alternatively, one can view a strong-seeded extractor as a family of functions, indexed by the seed, such that, for any given source *X*, most functions in the family output nearly uniform bits.

Using the probabilistic method, it is known that a random function is a strong-seeded extractor for d=log(n−k)+2log(1/ϵ)+O(1) and m=k−2log(1/ϵ)−O(1). Further, there are excellent explicit constructions of seeded extractors [12,13,14] that match the random construction (up to constants).

Recently, there has been exciting progress in the area of randomness extraction in two directions:

- *Robust variants of seeded extractors.* Motivated by applications in cryptography, a novel strengthening of the seeded extractor, called a non-malleable extractor (defined formally later), was introduced by Dodis and Wichs [15]. In particular, they proved that such non-malleable extractors would lead to important progress on a central problem in cryptography, known as privacy amplification [16,17,18,19]. Further variants of non-malleable extractors [20] have been shown to have application to the theory of error-correcting codes [21].
  Very informally, a non-malleable extractor nmExt can be thought of as having the guarantee that the output of nmExt looks uniform even if an adversary has access to its output on a different seed. This ‘pairwise independence’ property of the output of the extractor indeed turned out be a non-trivial challenge, as we discuss in later sections of this article.
- *Seedless extraction.* Recall that merely having a guarantee on the min-entropy of *X* is not sufficient for ensuring the possibility of producing truly random bits from *X* using an extractor. An important direction of investigation has been to study randomness extraction assuming further structure in the defective source *X*. This has seen a lot of progress in recent times, and we briefly discuss three major classes of sources:
  1. Independent sources: This is one of most well-studied models of weak sources, and makes a natural assumption that the extractor has access to multiple independent sources of randomness. There has been remarkable progress in constructing randomness extractors for such sources, leading to efficient construction close to the probabilistic bounds [22,23,24,25,26,27,28].
  2. Affine sources and extensions: In this setting, one imposes an algebraic structure on the support of the source and assumes that it is a vector space (on a finite field) of dimension *k*. It turns out that constructing such affine extractors has a variety of applications to cryptography [9,29,30] and complexity theory [31]. Starting with the early work of Gabizon and Raz [32] and Bourgain [33], there has been a lot of recent progress in constructing explicit affine sources that come very close to the optimal (non-explicit) construction [34,35,36,37,38]. This model of affine sources has been extended and studied in many different ways with important applications.
  3. Samplable sources: Another natural means of restricting the class of sources is to impose computational limitations on the algorithm that is producing the defective source. This direction of randomness extraction was initiated by Trevisan and Vadhan [39], and has seen very interesting progress recently [38,40,41,42,43]. In particular, we will discuss the setting of small-space sources, where the sampling algorithm is constrained to have limited space.

We conclude by noting that while the two directions seem unrelated superficially, it turns out that progress on explicitly constructing robust variants of seeded extractors played a key role in the advances in seedless extraction. In the sections below, we discuss these directions (robust seeded extractors and seedless extraction) in more detail.

## 2. Non-Malleable Extractors

A robust variant of a seeded extractor, known as a non-malleable extractor, was introduced by Dodis and Wichs [15], with applications to an important problem in cryptography known as privacy amplification [16,17,18,19].

Recall that an input $x\in {\{0,1\}}^{d}$ is a fixed point of a function $f:{\{0,1\}}^{d}\to {\{0,1\}}^{d}$ if f(x)=x). We are now ready to formally define non-malleable extractors.

**Definition** **3** (Non-Malleable Extractor)**.** *An (n,k)-(seeded) non-malleable extractor ${\mathrm{nmExt}:\{0,1\}}^{n}\times {\{0,1\}}^{d}\to {\{0,1\}}^{m}$ with seed length d and error ϵ satisfies the following: for any tampering function $f:{\{0,1\}}^{d}\to {\{0,1\}}^{d}$ with no fixed points, and any (n,k)-source X, and we have*

$$|\left(\mathrm{nmExt}\left(X,U_{d}\right),\mathrm{nmExt}\left(X,f\left(U_{d}\right)\right),U_{d}\right)-\left(U_{m},\mathrm{nmExt}\left(X,f\left(U_{d}\right)\right),U_{d}\right)|\leq \epsilon .$$

Dodis and Wichs [15] used a clever probabilistic argument to show the existence of non-malleable extractors with d=log(n−k)+2log(1/ϵ)+O(1) and m=k/2−log(1/ϵ)−logd−O(1), and left it open to explicitly construct non-malleable extractors.

The first explicit construction of a non-malleable extractor was given by Dodis, Li, Wooley and Zuckerman [44], and the extractor required min-entropy at least (1/2+δ)n, for any constant δ>0. Cohen, Raz and Segev [45] improved the output length of the non-malleable extractor, but still required the min-entropy rate to be more than half. Subsequently, Li [46] lowered the min-entropy requirement to (1/2−γ)n, for some tiny constant γ>0.

For a while, it appeared difficult to construct non-malleable extractors that work for significantly smaller min-entropy. Chattopadhyay, Goyal and Li [47] obtained an exponential improvement and constructed an explicit non-malleable extractor that worked for min-entropy poly(logn). Subsequently, an impressive line of work [23,26,27,28] has led to near-optimal constructions of non-malleable extractors. The best known construction is the following result obtained by Li [28].

**Theorem** **1** ([28])**.** *There exists a constant C>0 and an explicit (k,ϵ)-non-malleable extractor ${\mathrm{nmExt}:\{0,1\}}^{n}\times {\{0,1\}}^{d}\to {\{0,1\}}^{m}$, with min-entropy requirement $k\geq C(\log\log n+\log\left(1/\epsilon \right)\cdot {\left(\log\log\left(1/\epsilon \right)\right)}^{1-o(1)})$ and seed length $d=O(\log n+\log\left(1/\epsilon \right)\cdot {\left(\log\log\left(1/\epsilon \right)\right)}^{1-o(1)})$.*

A stronger variant of non-malleable extractors, called a *t*-non-malleable extractor, has been studied in the literature. This notion was introduced in [45], and such a *t*-non-malleable extractor nmExt satisfies the following property: let *X* be an (n,k)-source, and $f_{1},\ldots ,f_{t}$ be arbitrary tampering functions acting on *d* bits, with the restriction that the $f_{i}$s do not have fixed points. Then,

$$|\mathrm{nmExt}\left(X,U_{d}\right),{\left\{\mathrm{nmExt}\left(X,f_{i}\left(U_{d}\right)\right)\right\}}_{i\in [t]},U_{d}-U_{m},{\left\{\mathrm{nmExt}\left(X,f_{i}\left(U_{d}\right)\right)\right\}}_{i\in [t]},U_{d}|\leq \epsilon .$$

The non-malleable extractor in [47], and follow-ups, in fact satisfy this stronger notion. *t*-non-malleable extractors have played a key role in explicit constructions of extractors for independent sources. We discuss this later.

### 2.1. Correlation Breakers

A key definition that led to this impressive progress in constructions of non-malleable extractors is that of a correlation breaker with advice. To motivate this definition, consider the following natural scenario: Suppose that $Y_{1},Y_{2}$ are random variables that are arbitrarily correlated with each other, with the guarantee that $Y_{1}$ is uniformly distributed. Is it possible to take some additional amount of randomness to break the ‘correlation’ between these random variables? For example, one can consider the situation where we are given access to an independent (n,k)-source *X*, and we would like to construct a function CB such that $CB(X,Y_{1})$ is close to uniform, even conditioned on the joint random variable $CB(X,Y_{2})$. Clearly this is impossible in this generality since it could be the case that $Y_{1}=Y_{2}$. However, it turns out that if we allow the correlation breaker with an additional ‘advice string’, then one can define a meaningful notion. We define this notion formally below.

**Definition** **4** (Advice Correlation Breaker)**.** *A function ${\mathrm{ACB}:\{0,1\}}^{n}\times {\{0,1\}}^{d}\times {\{0,1\}}^{r}\to {\{0,1\}}^{m}$ is called an (k,ϵ)-advice correlation breaker if it satisfies the following:*

- *Let $Y_{1},Y_{2}$ be arbitrarily correlated random variables on ${\{0,1\}}^{d}$ such that, for some i∈{1,2}, we have $Y_{1}$ as the uniform distribution on ${\{0,1\}}^{d}$*
- *Let $\alpha_{1},\alpha_{2}\in {\{0,1\}}^{r}$ be advice strings, with the guarantee that they are distinct, then*
  $$|\left(\mathrm{ACB}\left(X,Y_{1},\alpha_{1}\right),\mathrm{ACB}\left(X,Y_{2},\alpha_{2}\right),Y_{1},Y_{2}\right)-\left(U_{m},\mathrm{ACB}\left(X,Y_{2},\alpha_{2}\right),Y_{1},Y_{2}\right)|\leq \epsilon .$$

The notion of an advice correlation breaker, was implicitly used in the works of Li [48], Cohen [49] and Chattopadhyay, Goyal and Li [47], and was explicitly defined by Cohen [50]. In particular, [47] used explicit constructions of advice correlation breakers to obtain an exponential improvement in terms of the min-entropy requirement of non-malleable extractors. We note that in the literature, a more general definition is considered where multiple tamperings are allowed, and further both X and Y are allowed to be tampered independently. For simplicity, we focus on the simpler definition here.

A particularly elegant construction of an advice correlation breaker in the case of r=1 (i.e., 1 bit of advice), called the flip-flop primitive, was given by Cohen [49] by employing the method of alternating extraction [51].

**Theorem** **2** ([49])**.** *There exists an explicit (k,ϵ)-advice correlation breaker $\mathrm{FF}:{\{0,1\}}^{n}\times {\{0,1\}}^{d}\times \{0,1\}\to {\{0,1\}}^{m}$ where k≥C(m+log(n/ϵ))), for some constant C>0 and d=O(log(n/ϵ)).*

For larger advice lengths, [47] constructed advice correlation breakers by ‘chaining’ together a group of such flip-flop primitives in a sequential manner. In particular, they obtained the following theorem.

**Theorem** **3** ([47])**.** *There exists an explicit (k,ϵ)-advice correlation breaker $\mathrm{ACB}:{\{0,1\}}^{n}\times {\{0,1\}}^{d}\times {\{0,1\}}^{r}\to {\{0,1\}}^{m}$ where k≥Cr(m+log(n/ϵ)), for some constant C>0 and d=O(rlog(n/ϵ)).*

We refer the reader to a recent survey [52] for a detailed demonstration of the construction of the flip-flop primitive and advice correlation breaker in the above theorem.

### 2.2. Advice Generators

A second key definition that has been crucial in constructing non-malleable extractors is that of an advice generator. Informally, this is described as follows: Suppose that we know that $Y_{1}$ and $Y_{2}$ are correlated random variables with the guarantee that $Y_{1}\neq Y_{2}$ (more formally, we assume that, for any *y*, $Pr[Y_{2}=y|Y_{1}=y]=0$); given access to an independent weak source *X*, can we generate advice strings $\alpha_{i}=\left(X,Y_{i}\right)$ such that $\alpha_{1}\neq \alpha_{2}$ with high probability? Clearly, if we do not restrict the lengths of the $\alpha_{i}$s, one can simply ignore *X* and define $\mathrm{AdvGen}(X,Y_{i})=Y_{i}$. To make advice generators useful for constructing non-malleable extractors, it turns out that we must insist the advice generated to be much shorter than the lengths of the $Y_{i}$s, which makes it more non-trivial to construct.

We now formally define advice generators.

**Definition** **5** (Advice Generators)**.** *An (k,ϵ)-advice generator $\mathrm{AdvGen}:{\{0,1\}}^{n}\times {\{0,1\}}^{d}\to {\{0,1\}}^{r}$ satisfies the following guarantee: Let $Y,Y^{\prime }$ be correlated random variables on ${\{0,1\}}^{r}$, such that Y follows the uniform distribution on ${\{0,1\}}^{r}$. Further assume the property that, for any y in the support of Y, $Pr[Y^{\prime }=y|Y=y]=0$. Let X be an (n,k)-source that is independent of $\left(Y_{1},Y_{2}\right)$. Then, with probability 1−ϵ over the fixing of $Y_{1},Y_{2},\mathrm{AdvGen}(X,Y_{2})$, we have*

$$\mathrm{AdvGen}(X,Y_{1})\neq \mathrm{AdvGen}(X,Y_{2}).$$

Optimal constructions are known for advice generators.

**Theorem** **4** ([24,47])**.** *There exist constants $C,C^{\prime }$ and an explicit (k,ϵ)-advice generator $\mathrm{AdvGen}:{\{0,1\}}^{n}\times {\{0,1\}}^{d}\to {\{0,1\}}^{r}$ with k≥Clog(n/ϵ), $d=C^{\prime }\log\left(n/\epsilon \right)$ and a=O(log(1/ϵ)).*

The basic idea behind constructing such advice generators is quite simple: we know that $Y_{1}\neq Y_{2}$, and hence they must differ at some coordinate. We first transfer this ‘worst-case guarantee’ into an ‘average-case guarantee’ by encoding the $Y_{i}$s using an asymptotically good error correcting code. This ensures that the encoded strings differ on a constant fraction of the coordinates. Now, one can use sampling techniques based on seeded extractors [53] to output a small pseudorandom set of coordinates with the guarantee that they strongly differ (with high probability).

### 2.3. Combining Advice Correlation Breakers and Advice Generators

We now briefly sketch the high-level ideas of constructing non-malleable extractors by combining the two definitions. Recall that our setting is the following: *X* is an (n,k)-source, and *Y* is a uniform seed of length *d*, which is tampered by a function *f* that has no fixed points. Let us define $Y_{1}=Y$, and $Y_{2}=f\left(Y\right)$. A natural strategy to construct a non-malleable extractor, given the above primitives, is the following:

- Define $\alpha_{i}=\mathrm{AdvGen}\left(X,Y_{i}\right)$, where AdvGen is an (k,ϵ)-advice generator.
- Define $Z_{1}=\mathrm{nmExt}\left(X,Y_{1}\right)=\mathrm{ACB}\left(X,Y,\alpha_{1}\right)$, where ACB is an (k,ϵ)-advice correlation breaker.

It turns out that the above construction actually works, but one needs to deal with a few subtle issues: note that the advice string is correlated with the source *X* and the seed *Y*, where, as in the definition of the advice correlation breaker, we assume that the advice strings are fixed.

To deal with the above issue, one insists on a stronger property from the advice generator that it is possible to fix the advice strings without losing independence of the source *X* and the seeds $Y_{1},Y_{2}$. Further, we must ensure that, on fixing the advice strings, *X* and *Y* do not lose too much min-entropy. The latter issue is addressed by the fact that the advice strings are much shorter than the seed (or the entropy in *X*). The former issue requires one to carefully look into the construction of the advice generator and carefully fix the random variables while ensuring that no dependencies are created between *X* and $\left(Y_{1},Y_{2}\right)$.

## 3. Seedless Extraction

We now turn our attention to the setting of randomness extraction where the extractor no longer has access to an independent seed. However, recall that it is impossible to extract randomness from a general (n,k)-source, and hence one must assume more structure on the source to enable randomness extraction. We now discuss various directions of research that have been considered.

### 3.1. Extractors for Independent Sources

A particularly well-studied model of weak sources is the independent source setting. Here, one assumes that the extractor has access to *C* independent (n,k)-sources $X_{1},\ldots ,X_{C}$, for some C≥2. The most ambitious setting is that of C=2, and it is conceivable that the task of extraction becomes easier as *C* grows larger.

We formally define a C-source extractor.

**Definition** **6.** *A (C,k,ϵ)-source extractor $\mathrm{Ext}:{\left({\{0,1\}}^{n}\right)}^{C}\to {\{0,1\}}^{m}$ satisfies the following guarantee: let $X_{1},\ldots ,X_{C}$ be independent (n,k)-sources. Then, $|\mathrm{Ext}\left(X_{1},\ldots ,X_{C}\right)-U_{m}|\leq \epsilon$.*

A probabilistic argument shows that a random function is a 2-source extractor for min-entropy logn+O(1) (for constant output length and constant error). The problem of explicitly constructing a 2-source extractor was raised by Chor and Goldreich [9], where they constructed an extractor for min-entropy (0.5+δ)n, for any constant δ>0. This was slightly improved by Bourgain [54] to achieve min-entropy (1/2−γ)n, for some tiny constant γ>0. It remained a challenging open problem to obtain further improvements until the work of Chattopadhyay and Zuckerman [55], who gave an explicit 2-source error construction for min-entropy poly(logn). A recent line of work [22,23,24,25,26,27,28] further lowered the min-entropy to $\log n\cdot {\left(\log\log n\right)}^{1-o(1)}$, thus coming very close to optimal bounds.

Since the problem of constructing 2-source extractors appeared challenging for a while, several researchers developed methods to construct extractors for *C*-independent sources [30,48,56,57,58]. In particular, Li [58] obtained a near-optimal 3-source extractor that works for poly(logn) min-entropy and has negligible error.

We now give a sketch of the 2-source extractor construction in [55], which set up a framework that has been used and refined to obtain all the further improvements.

#### 3.1.1. Explicit 2-Source Extractors

Consider the setting in which we have two independent (n,k)-sources *X* and *Y*. As a first attempt, consider taking a (k,ϵ)-strong-seeded extractor $\mathrm{Ext}:{\{0,1\}}^{n}\times {\{0,1\}}^{d}\to \{0,1\}$, and for any seed $s\in {\{0,1\}}^{d}$, define $f_{s}\left(X\right)=\mathrm{Ext}\left(X,s\right)$. It follows from the definition of a strong-seeded extractor that, for most (i.e., $1-\sqrt{\epsilon }$ fraction) of the seeds, $f_{s}\left(X\right)$ is close to a uniform bit. Further note that d=O(logn) (for $\epsilon =1/n^{O(1)}$), and hence one could enumerate over all the seeds in polynomial time to produce a source *Z* of length $D=2^{d}=poly\left(n\right)$, where $Z_{s}=f_{s}\left(X\right)$. However, it is not clear how to push forward with this approach since the bits in $Z_{s}$ could be correlated in arbitrary ways (even though most of the bits are uniform).

The strategy in [55] is to perform the above step using a *t*-non-malleable extractor (discussed above in the section on non-malleable extractors), for some parameter *t*, which is set to poly(logn). The key result that is shown in [55] is that the resulting source *Z* that is obtained by enumerating over the seeds satisfies the following property: there is a large fraction of coordinates, given by a set *S*, such that, for any subset T⊂S, |T|=t, the source $Z_{T}$ obtained by projecting *Z* to the coordinates in *T* is close to the uniform distribution $U_{t}$. In other words, $Z_{S}$ is an almost *t*-wise independent distribution, and the coordinates of *Z* that are outside *S* depend arbitrarily on $Z_{S}$. For technical reasons (related to the fact that $Z_{S}$ follows an almost *t*-wise independent distribution rather than a *t*-wise independent), it can be shown that it is impossible to extract from *Z*. This is exactly where the second source *Y* comes in, and the main use of this source is to pseudorandomly sample a subset of coordinates of *Z*. In particular, the following theorem is proven in [55].

**Theorem** **5.** *There exists a function, ${\mathrm{Reduce}:\{0,1\}}^{n}\times {\{0,1\}}^{n}\to {\{0,1\}}^{m}$, m=poly(n), such that, if X and Y are independent (n,poly(logn))-sources, then Z=Reduce(X,Y) is $1/n^{\omega (1)}$-close to a source $Z^{\prime }$ that satisfies the following: there exists a subset S⊂[m], $\left|S|\geq m(1-m^{\delta }\right)$, for some δ>0, such that $Z_{S}^{\prime }$ is t-wise independent, for some t=poly(logn).*

It turns out that extraction from such sources is related to collective coin flipping [59], a problem that is well studied in distributed computing. We first define a class of sources that captures the output of the reduce function.

**Definition** **7** (Non-Oblivious Bit-Fixing (NOBF) sources)**.** *A distribution Z on ${\{0,1\}}^{m}$ is called a (q,t)-NOBF source if there exists a set S⊂[m], such that |S|≥q, and $Z_{S}$ is t-wise independent (i.e., projection of $Z_{S}$ to any t coordinates is the distribution $U_{t}$).*

Bit-fixing sources have been studied intensely in randomness extraction, with the simplest setting of oblivious bit-fixing sources corresponding to a source with an unknown set of independent and uniform coordinates (and the rest are fixed to constants that do not depend on the random values). The NOBF sources defined above correspond to a much trickier setting because of two reasons: (i) the bad bits (outside *S*) can depend on the values of the good bits (coordinates in *S*); (ii) the coordinates in *S* are not independent and uniform, but only satisfy the weaker guarantee of *t*-wise independence.

In fact, Viola [41] proved that the MAJORITY function is an extractor for such NOBF sources in the setting of t=O(1) and $q\geq m-m^{1/2-\gamma }$, for any constant γ>0. However, the δ in Theorem 5 is a tiny constant, much smaller than 1/2 (in fact, one can provably show that the method used in [55] cannot yield δ≥1/2), and hence the MAJORITY function cannot be used.

More generally, a natural question is to investigate the property of a Boolean function $f:{\{0,1\}}^{n}\to \{0,1\}$ that allows it to extract from NOBF sources. It turns out that this is exactly captured by the notion of resilient functions. Informally, an (r,ϵ)-resilient function $f:{\{0,1\}}^{n}\to \{0,1\}$ satisfies the property that any subset *T* of variables of size at most *r* cannot bias the function *f* by much. More formally, define the influence of a set *T* on *f* as the probability that *f* is not fixed on sampling variables outside *T*. Usually, it is assumed that the variables outside *T* are sampled from the uniform distribution. However, in our application, it is useful to define influence assuming that the variables outside *T* are sampled from a *t*-wise independent distribution. Then, we can define a (t,r,ϵ)-resilient function as follows: for any subset *T* of variables of size at most *q*, the influence of *T* (when variables outside *T* are sampled from an *r*-wise independent distribution) is bounded by ϵ.

Using the notion of resilient functions, one can state Viola’s result [41] as showing that MAJORITY is a $\left(O\left(1\right),n^{1/2-\gamma },\epsilon \right)$-resilient function, for any γ>0 and $\epsilon =1/t^{1/2-o(1)}$.

An important contribution of [55] was to construct a $\left(poly\left(\log n\right),n^{1-\delta },1/n^{\Omega (1)}\right)$-resilient function based on a derandomization of a probabilistic construction of Ajtai and Linial [60], also relying on the fact that polylog-wise independent distributions fool constant depth circuits [61]. Using such resilient functions, it is easy to extract from the NOBF source produced by Theorem 5.

An improved reduction from two independent sources to an NOBF source was subsequently obtained by Ben-Aroya, Doron and Ta-Shma [22] that allowed the use of MAJORITY as the extractor for the NOBF source. This played a key role in obtaining near-optimal two-source extractors (in terms of min-entropy).

It still remains a challenging open problem to construct negligible error two-source extractors for min-entropy that is significantly smaller than n/2. In particular, it is not difficult to show that extractors for NOBF sources cannot have negligible error, and hence one way of making progress in this direction will be to find methods of removing the use of such extractors in two-source extractor constructions. One such approach, which is related to constructing improved *t*-non-malleable extractors, is proposed in [62].

#### 3.1.2. Beyond Independent Source Setting

Several recent works [63,64,65,66] have considered the models of weak sources that extend beyond the independent source model. In [65], the authors explore the setting where some of the independent sources are faulty and contain no min-entropy, and a stronger variant where the faulty sources are allowed to depend on a few of the good sources. In [63], the authors study an even stronger setting where a faulty source can depend on all sources that have been sampled before it. In this model, they prove impossibility results on randomness extraction and obtain somewhere extractors. In [64], among various settings that are studied, they study the problem of randomness extraction between two-sources by assuming a bound on the correlation (in various information theoretic measures such as mutual information and cross-influence). In [66], the task of randomness extraction is studied when there is joint leakage on a bounded number of the independent sources, and applications of such extractors are shown in constructing leakage-resilient secret-sharing schemes and in obtaining average-case lower bounds against bounded-collusion communication protocols.

### 3.2. Extractors for Affine Sources

Another well-studied model of weak sources is affine sources, which are defined as follows.

**Definition** **8** (Affine source)**.** *Fix a finite field F. An ${\left(n,k\right)}_{q}$ affine source X is a distribution that is uniform over some unknown subspace of dimension k in $F^{n}$.*

Apart from being a natural question, affine extractors (over $F_{2}$) have found applications in cryptography [9,29,30] and circuit lower bounds [31]. Further, it turns out that affine extractors can be employed to extract from various other models of weak sources (see [37] for more discussion).

Gabizon and Raz [32] considered the problem of extracting randomness from affine sources over large fields, and in fact constructed an extractor that works for k≥1, assuming the field size q=poly(n). It turned to be much more challenging to extract randomness in the small field setting, and in particular, q=2 seemed to be the most difficult. Bourgain [33] constructed an affine extractor for min-entropy Ω(n) and negligible error, and this was slightly improved to $\Omega (n/\sqrt{\log\log n})$ by Yehudayoff [34] and Li [35]. Finally, a major improvement was obtained by Li [36], where an affine extractor was obtained for poly(logn) min-entropy (and polynomially small error). This was recently sharpened by Chattopadhyay, Goodman and Liao [37], where they obtained an affine extractor for min-entropy $\left(\log n\right)\cdot {\left(\log\log n\right)}^{1+o(1)}$ (and constant error).

The extractor construction by Li [36] (and subsequently [37]) follows a strategy that is similar to the [55] framework of extracting randomness from two independent sources. In particular, Li [57] showed a means of transforming an affine source into an NOBF source and using known extractors for NOBF sources. The reduction of an affine source into an NOBF source relies on stronger variants of correlation breakers, and a key ingredient in their construction is linear seeded extractors (i.e., seeded extractors that additionally satisfy the guarantee that, for any fixing of the seed, the extractor is a linear function of the source). The further improved affine extractor in [37] is obtained by adapting ideas used in constructions of (standard) correlation breakers to the affine setting. A recent follow-up work by Chattopadhyay and Liao [38] gives a black-box way of using standard correlation breaker to construct correlation breakers in the affine setting.

### 3.3. Extractors for Sumset Sources

Chattopadhyay and Li [42] considered a general model of weak sources called sumset sources, which are defined as follows.

**Definition** **9.** *An (n,k,C)-sumset source X is of the form $X_{1}+X_{2}+\ldots +X_{C}$, where the $X_{i}$s are independent (n,k)-sources.*

Clearly, an extractor for (n,k,C)-sumset source can extract from the model of *C* independent sources (by simply applying the extractor on the sum of the sources). In fact, such a sumset source works for the more general model of an *C*-interleaved source defined as an unknown interleaving of *C* independent sources. Further, note that an affine source *X* (over $F_{2}$) of min-entropy *k* can be expressed as the sum of *C* independent affine sources, each of entropy k/C. Finally, in the next section, we will see that such sumset sources have applications to extracting randomness from sources that are sampled by algorithms with limited memory. Thus, the model of sumset sources captures many well-studied models of weak sources and in fact extends them. In fact, unlike the other weak sources considered so far, it is not even clear if a random function is an extractor for sumset sources (see [38] for more on this).

Ref. [42] constructed an extractor for (n,k,C)-sumset source, with k=poly(logn) and a large enough constant *C*. This result was recently improved by Chattopadhyay and Liao [38] to obtain an extractor (with polynomially small error) in the setting of C=2, and k=poly(logn). Prior to the work of [38], the only known extractor for the sum of two sources was the Paley graph extractor [9], which required one of the sources to have min-entropy at least (1/2+δ)n, for any constant δ>0.

Both of these constructions [38,42] use the framework of [55], and obtain reductions from sumset sources to NOBF sources.

### 3.4. Extractors for Small-Space Sources

A line of work initiated by Trevisan and Vadhan [39] investigates the problem of extracting randomness from a weak source, assuming that it was sampled by a low-complexity algorithm. In this direction, Kamp, Rao, Vadhan and Zuckerman [40] introduced the notion of small-space sources, which are distributions sampled by an algorithm with limited memory. One can view such a source as being generated by taking a random walk on a (layered) branching program of width $2^{s}$ (where *s* corresponds to the space of the algorithm), where each edge is labeled with a bit b∈{0,1} and a transition probability. Such sources are called small-space sources and are known to capture a variety of other well-studied models of weak sources. We refer the reader to [40] for a formal definition and discussion on applications to extracting from other models of defective sources.

Non-constructively, it was shown in [40] that a random function is an extractor for a space-*s* source with entropy k≥2s+O(log(n/ϵ)) and error ϵ. Further, [40] gave an explicit extractor construction for min-entropy $O(n^{1-\gamma }s^{\gamma })$ (and negligible error), thus leaving a large gap between the existential and explicit constructions. Using improved extractors for the robust variant of independent sources (called adversarial sources [65]), Ref. [43] improved the min-entropy requirement to roughly $n^{1/2+\delta }s^{1/2-\delta }$, for any constant δ>0, while obtaining negligible error.

In the setting of polynomially small error, [42] obtained a reduction from small-space to sumset sources, and improved the min-entropy requirement to $s^{1.1}\cdot 2^{{\left(\log n\right)}^{1/2+\delta }}$, for any δ>0.

A new reduction from small-space sources to affine sources was given by Chattopadhyay and Goodman [43].

**Theorem** **6.** *Let X be an (n,k)-source sampled by a space-s algorithm. Then, X is $2^{-\Omega (k)}$-close to a convex combination of affines sources with min-entropy Ω(k/slog(n/k)).*

Thus, when using explicit affine extractors, the min-entropy requirement of small-space source extractors was further improved to s·poly(logn) in [43] by a new reduction to affine sources.

Very recently, [38] obtained an extractor for space-*s* sources with min-entropy requirement k≥2s+poly(logn). The result in [38] is based on the following reduction from small-space sources to 2-sumset sources (i.e., the sum of two independent sources).

**Theorem** **7.** *Let X be an (n,k)-source sampled by a space-s algorithm. Then, X is ϵ-close to a convex combination of $\left(n,k^{\prime },2\right)$-sumset sources, where $k^{\prime }\geq k/2-s-\log\left(n/\epsilon \right)-O\left(1\right)$.*

This gives an optimal dependence on *s* since it is shown in [40] that it is impossible to extract from space-*s* sources when k<2s.

## 4. Conclusions and Open Questions

There has been very interesting progress in explicit constructions of randomness extraction in two major directions: (i) robust variants of seeded extractors known as non-malleable extractors; (ii) seedless extraction for various models of weak sources such as independent sources, affine sources, sumset sources and small-space sources. As we saw, progress in (ii) was critically dependent on progress and techniques from (i), and, in particular, on constructions of objects known as correlation breakers. Further, such robust variants of seeded extractors are of independent interest and have found many applications in cryptography, such as privacy amplification, robust secret-sharing schemes, etc.

Major questions that remain open are low-error constructions of extractors for various models of seedless extraction (with a central problem being the two-independent-source setting). Further, a major idea in designing seedless extractors for various models of is to find reductions to NOBF sources and use known extractors for NOBF sources. This presents a bottleneck in obtaining low error, since it is known that extractors for NOBF sources cannot have negligible error [67]. Thus, a natural direction of future investigation is to identity simple classes of sources that permit low-error extraction, and find new reductions from models such as two independent sources (or affine sources) to this simpler model.

Finally, another interesting direction is to find more applications in complexity theory and cryptography of the recent advances made in explicit constructions of extractors. For example, as mentioned above, the best known explicit circuit lower bounds are against affine extractors [31]. Can sumset extractors be leveraged to achieve further progress?

## Data Availability

Not applicable.
